# Aerobic Oxidative Desulfurization by Supported Polyoxometalate Ionic Liquid Hybrid Materials via Facile Ball Milling

**DOI:** 10.3390/molecules29071548

**Published:** 2024-03-29

**Authors:** Qian Wang, Tianqi Huang, Shuang Tong, Chao Wang, Hongping Li, Ming Zhang

**Affiliations:** 1 Institute for Energy Research, Jiangsu University, Zhenjiang 212013, China; wangq@zhensheng.net.cn (Q.W.); 18652885629@163.com (T.H.); shuangtong0923@163.com (S.T.); chaowang@ujs.edu.cn (C.W.); hongpingli@ujs.edu.cn (H.L.); 2 Hangzhou Zhensheng Technology Co., Ltd., Hangzhou 311100, China

**Keywords:** polyoxometalate, ionic liquid, aerobic oxidation desulfurization, ball milling, solvent free

## Abstract

With the increasingly strict limitations on emission standards of vehicles, deep desulfurization in fuel is indispensable for social development worldwide. In this study, a series of hybrid materials based on SiO_2_-supported polyoxometalate ionic liquid were successfully prepared via a facile ball milling method and employed as catalysts in the aerobic oxidative desulfurization process. The composition and structure of prepared samples were studied by various techniques, including FT-IR, UV-vis DRS, wide-angle XRD, BET, XPS, and SEM images. The experimental results indicated that the synthesized polyoxometalate ionic liquids were successfully loaded on SiO_2_ with a highly uniform dispersion. The prepared catalyst (C_16_PMoV/10SiO_2_) exhibited good desulfurization activity on different sulfur compounds. Moreover, the oxidation product and active species in the ODS process were respectively investigated via GC-MS and ESR analysis, indicating that the catalyst can activate oxygen to superoxide radicals during the reaction to convert DBT to its corresponding sulfone in the fuel.

## 1. Introduction

The rapid growth of the population has brought significant improvements in urbanization and industrialization, leading to a continuous increase in energy consumption, such as fossil fuels [1,2,3]. However, the combustion of these fuels containing various kinds of sulfur compounds including thiol, thioether and thiophene would result in the emission of SO_x_, which is the main source of environmental pollution issues (e.g., acid rain, haze and smog), severely impacting environmental ecology and human health [4,5]. As a result, many countries have implemented stricter regulations and fuel standards to limit the sulfur content in fuel. With the improvement in regulations, the sulfur content is now limited to less than 10 ppm and will gradually move towards being sulfur free in the future [6,7,8]. As the conventional desulfurization technology in the petroleum refining industry, hydrodesulfurization (HDS) can easily remove the aliphatic sulfur compounds including thiol, thioether and disulfide, but it is not effective for the removal of aromatic sulfur compounds (e.g., dibenzothiophene and its derivatives) [9,10,11]. To reach deep desulfurization on aromatic sulfur compounds in the HDS process, higher operation temperatures and pressures are required, inevitably increasing the production costs of fuel oil [6,12,13]. In recent years, various non-HDS technologies have been explored to achieve the goal of deep desulfurization, including oxidative desulfurization (ODS), extractive desulfurization (EDS), adsorptive desulfurization (ADS) and biological desulfurization (BDS).

Among them, oxidative desulfurization (ODS) is considered as a green and effective method for the removal of aromatic sulfur compounds such as dibenzothiophene (DBT) from fuel at room temperature and atmospheric pressure [14,15,16]. In the ODS process, the organosulfur compounds could be converted to their sulfoxides or sulfones by some oxidants in the presence of a catalyst and then removed from the oil phase via adsorption or extraction [10,17]. In addition, compared to other desulfurization methods, oxidative desulfurization can achieve deep or even complete desulfurization. Moreover, the choice of oxidant and catalyst is also crucial for the desulfurization performance. Various kinds of oxidants have been explored in the ODS process, including hydrogen peroxide (H_2_O_2_) [10,18,19], oxygen (O_2_) [15,20] and ozone [21]. In particular, air with abundant oxygen on the earth can be selected as a green and promising candidate for the ODS process. Unlike other oxidizers, oxygen will not produce additional products after reaction, being an environmentally friendly oxidizer [22].

In terms of catalysts, various kinds of materials have been extensively studied for the ODS process in recent years, such as metal-organic frameworks (MOFs) [23,24], metal oxides [25], ionic liquids (ILs) [26], polyoxometalates [27] and low-eutectic solvents [28,29]. Among these materials, ionic liquids (ILs) are a type of room-temperature molten salt made up of organic cations and inorganic anions and possess unique properties such as high polarity, low volatility, and strong designability; they have been employed as catalysts in different processes such as separation processes, electrochemical and chemical reactions in recent years [30,31]. Moreover, the introduction of active sites with functionalized roles in ionic liquids can facilitate the application of functional ionic liquids in different fields. Active site-functionalized ionic liquids can also effectively solve the problem of active site aggregation. In the ODS process, the ability to tailor anions and cations to construct ILs with a high affinity for sulfides allows for design flexibility with significant advantages. In recent years, functionalized polyoxometalate ionic liquids can be obtained by introducing metal salts as the active center into ionic liquids via ion exchange. However, limitations of ILs, such as low surface area and difficulty in separation after reaction, hinder their wide application in the desulfurization industry [32]. Nevertheless, loading ILs on a solid support can solve the problems above and reduce the amount of ILs to cut production costs [33,34,35]. The synergistic effect of ILs and solid supports capitalizes on the large specific surface area of the solid material. At the same time, the lipophilic nature of ILs enables the overall catalyst to remove sulfides from fuel without the addition of an extractant. Zhang et al. successfully prepared silica-supported polyoxometalate-based ionic liquids and achieved 100% removal efficiency for 4,6-DMDBT under mild conditions without using an organic solvent as an extractant [36,37,38,39,40,41]. 

This work successfully prepared a series of supported polyoxometalate ionic liquid materials via a facile ball milling method. The composition and structure of catalysts was studied in detail by various analytical techniques as well as their catalytic activity in the ODS process. The experimental results indicated that the uniform dispersion of active components within the polyoxometalate ionic liquids on the solid support material was crucial to promote the oxidation reaction. The effect of the ionic liquid-to-carrier ratio was investigated on the specific surface area and desulfurization activity of the catalyst. Moreover, the optimization of reaction conditions was investigated by adjusting the amount of oxidant for the desulfurization system, catalyst quality, reaction temperature, and reaction duration; the recycling performance of the catalyst was also evaluated. Under the optimized reaction conditions, the catalytic performance was evaluated upon removing various sulfur-containing compounds such as DBT, 4-MDBT and 4,6-DMDBT. Furthermore, the investigation on the reaction mechanisms of the ODS process was conducted by integrating ESR and GC-MS analysis.

## 2. Results and Discussion

### 2.1. Characterization of the Catalyst

**Figure 1:**
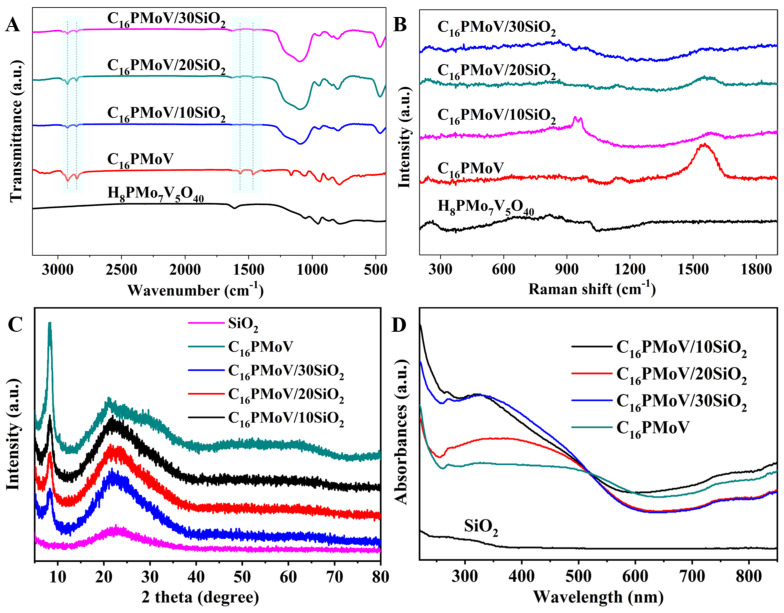
(**A**) FT-IR spectra, (**B**) Raman spectra, (**C**) XRD patterns and (**D**) UV-DRS spectra of different samples.

Figure 1A illustrates the FT-IR spectra of different samples. For the sample H_8_PMo_7_V_5_O_40_ catalyst, four classical bands ascribed to the Keggin structure can be found in the 600–1200 cm^−1^ range. The antisymmetric stretching vibration (776~808 cm^−1^) between the oxygen atom and the metal coordination atom (Mo-O_c_-Mo) was present in the MoO_6_ octahedron. The anti-symmetric stretching vibration near 872 cm^−1^ indicates the interplay between oxygen and metal coordination atoms in the MoO_6_ octahedron’s periphery. The bands at 937~945 cm^−1^ and near 1060 cm^−1^ correspond to the metal coordination anti-expansion vibration occurring between the atom and the terminal oxygen atom (Mo = O_t_) and the central oxygen atom (P-Oa) [42], respectively. With the exception of the sample H_8_PMo_7_V_5_O_40_, the peaks around 2923, 2853, 1570 and 1467 cm^−1^ observed in the remaining catalysts containing ionic liquids were assigned to the asymmetric stretching vibration of C-H, the stretching vibration of C-H, the C-N bending vibration, and the C-C bending vibration, respectively [43]. Moreover, the characteristic bands for the support SiO_2_ were also observed, including asymmetric vibrations of Si-O-Si [44] with bending vibrations at 1090 cm^−1^ and 460 cm^−1^ and O-Si-O [45] bonding at 795 cm^−1^. These results indicate that the ionic liquid C_16_PMoV was successfully introduced on silica and the preserved Keggin structure POM material after the ball milling process.

To further study the structural information of the prepared samples, Raman spectra were employed in this study (Figure 1B). For the sample H_8_PMo_7_V_5_O_40_, the specific peaks of the Keggin structure can be observed around 247, 864 and 987 cm^−1^, which is respectively ascribed to the vibration of νs(Mo-Oa), νas (Mo-Ob-Mo) and νs (Mo-Od) [46]. For ionic liquid C_16_PMoV, except for the peaks for the polyoxometalate structure, two distinct characteristic peaks around 1160 cm^−1^ and 1545 cm^−1^ can be observed, which are respectively attributed to the bending vibration of the C-H bond and the stretching vibration of the C=C bond in the imidazolium cation with a long carbon chain. Moreover, the characteristic peaks of the ionic liquid C_16_PMoV can still be observed in the IL-supported samples. These results further indicated the stability of the Keggin structure in the supported catalyst. XRD patterns of different samples are presented in Figure 1C. For the support SiO_2_, a broad diffraction around 2θ = 23° can be observed, ascribed to the amorphous structure. On the other hand, a typical peak for the Keggin structure around 2θ = 8° can be found for the IL (C_16_PMoV) and IL-supported materials [47], indicating the retention of the POM structure after the ball milling process. These results obtained from FT-IR, Raman and XRD verified that the synthesized functionalized ionic liquids were successfully loaded on SiO_2_ with the preserved Keggin structure by the ball milling method, validating the feasibility of this method with subsequent broader applications.

To study the local environment of transition metal in the sample, UV-vis diffuse reflectance spectra of various samples are depicted in Figure 1D. For the support SiO_2_, no apparent absorption can be found in the UV spectrum. Notably, a distinct absorption band around 280 nm is observed for the IL (C_16_PMoV) and IL-containing materials, indicating the charge transfer between the metal atom and the coordinating electron in the sample [48]. Additionally, the absorption band at 350 nm is attributed to the integration of vanadium into the Keggin structure [47]. The results also demonstrated the successful synthesis of polyoxometalate-based ionic liquid-based materials, consistent with the FT-IR spectra results. 

**Figure 2:**
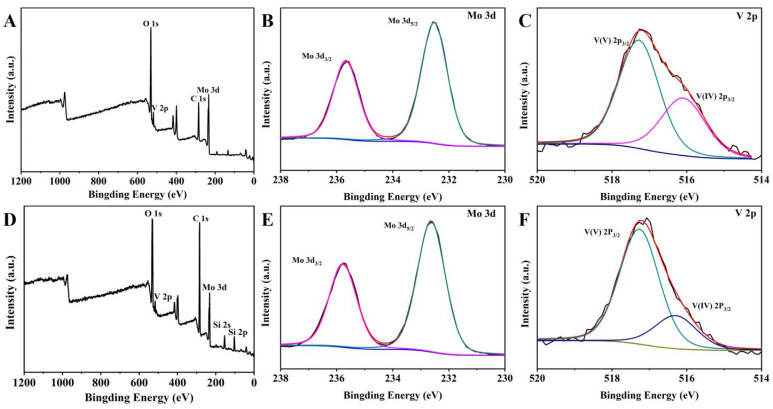
XPS analysis of C_16_PMoV: (**A**) survey, (**B**) Mo 3d, (**C**) V 2p and XPS spectra of C_16_PMoV/10SiO_2_: (**D**) full spectrum, (**E**) Mo 3d, (**F**) V 2p.

The elemental valence states and the surface chemical composition of C_16_PMoV and C_16_PMoV/10SiO_2_ were further investigated using XPS analysis (Figure 2). For C_16_PMoV, the binding energy peaks of C, O, Mo and V can be found, indicating the composition of the prepared ionic liquid (Figure 2B). The binding energies at 235.67 eV and 232.57 eV are respectively assigned to Mo 3d3/2 and Mo 3d5/2 (Figure 2C), indicating the presence of Mo^6+^ [49]. For the sample C_16_PMoV/10SiO_2_, the binding energies of Mo 3d3/2 and Mo 3d5/2 C_16_PMoV/10SiO_2_ emerged at 235.77 and 232.67 eV, respectively (Figure 2E). Compared to Mo 3d binding energy of C_16_PMoV, this shift indicates a decrease in the density of Mo^6+^ [50]. To further study the V 2p spectra of the samples, two peaks around 517.27 eV and 516.07 eV can be observed for IL C_16_PMoV (Figure 2C), which are respectively attributed to V^5+^ and V^4+^ [50,51]. After the immobilization of IL on the support (Figure 2F), a weakening of the V^4+^ peak and a shift toward higher intensities can be found. Evidently, the different binding energy in the C_16_PMoV/10SiO_2_ system presented a different reductive valence state during the loading process, indicating the change in chemical microenvironment of Mo and V. The outermost electrons of Mo and V may be affected by the electron shielding effect of SiO_2_, resulting in an increase in the overall electron binding energy [52]. 

**Figure 3:**
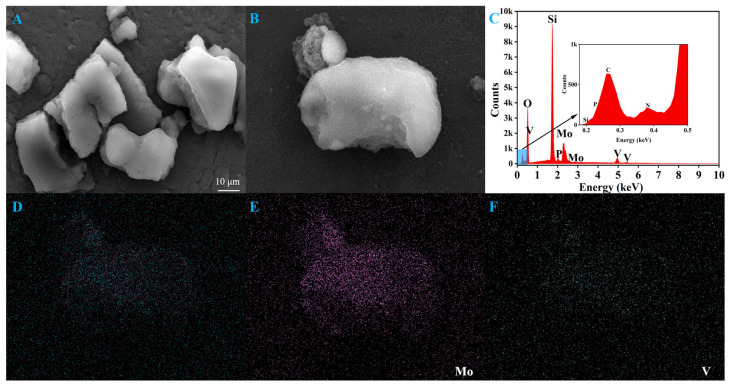
SEM images: (**A**) SiO_2_, (**B**) C_16_PMoV/10SiO_2_; EDS (**C**) of C_16_PMoV/10SiO_2_ and mapping images (**D**–**F**).

**Figure 4:**
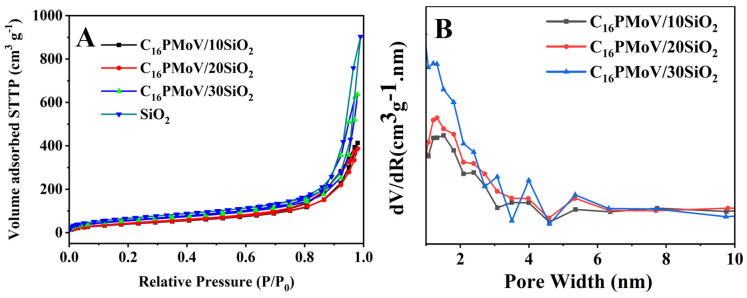
(**A**) Nitrogen adsorption-desorption isotherms and (**B**) pore size distributions of hybrid materials of different samples.

The morphology of the support SiO_2_ and the catalyst C_16_PMoV/10SiO_2_ were examined using SEM images (Figure 3). Compared to the pure SiO_2_ (Figure 3A), the SiO_2_ with IL still exhibits a bulk structure with a smooth surface, indicating no apparent changes in the support after the introduction of IL with the ball milling method. Furthermore, EDS mapping was performed to detect the main elemental composition of the sample (Figure 3C–F). Figure 3C illustrates the successful loading of the ionic liquid on SiO_2_. Mapping images in Figure 3E,F show that the Mo and V elements are evenly distributed in the sample, agreeing well with the XRD analysis results [53]. These results indicate that the ball milling process facilitated the successful preparation of hybrid materials with a uniform dispersion of IL C_16_PMoV, ensuring ample interaction between the active centers and the sulfide to the ODS process. N_2_ adsorption-desorption isotherms of various samples were conducted to investigate the textural structure of the prepared samples (Figure 4). The isotherms for all catalysts exhibited type IV isothermal adsorption lines with H1 hysteresis loop in the *p*/*p*_0_ range of 0.4–1.0 (Figure 4A), which could be attributed to the formation of stacked slit-type pores with a pore size distribution of 0.8–2.2 nm (Figure 4B) during the loading of the samples [54]. Besides, the specific surface area parameters of C_16_PMoV/nSiO_2_ and pure SiO_2_ are shown in Table 1. The specific surface area of pure SiO_2_ was the most extensive (447.9 m^2^/g). In contrast, the specific surface area of the catalysts obtained after the introduction of polyoxometalate ionic liquids on SiO_2_ was significantly decreased, and the specific surface area of the material C_16_PMoV/SiO_2_ was still beyond 250 m^2^/g. It is worth noting that the particular surface area gradually decreases with the increase in IL content, which is attributed to the fact that more IL is filled into the pores of the support during the preparation process.

### 2.2. Catalytic Activity

**Figure 5:**
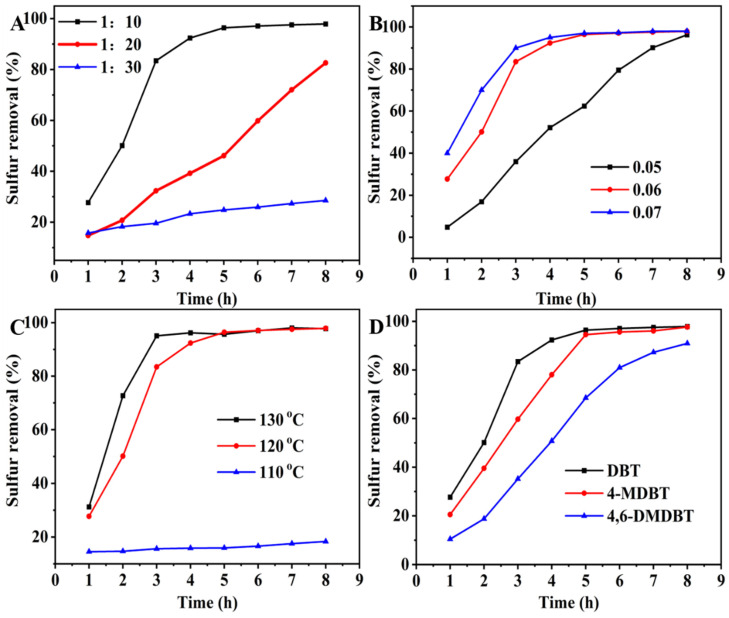
The effects of different factors: (**A**) different IL content, reaction conditions: m(catalysts) = 0.06 g, T = 120 °C, ν(air) = 100 mL/min; (**B**) catalyst mass, reaction conditions: T = 120 °C, ν(air) = 100 mL/min; (**C**) temperature, reaction conditions: m(catalyst) = 0.06 g, ν(air) = 100 mL/min; (**D**) sulfides, reaction conditions: m(catalysts) = 0.06 g, T = 120 °C, ν(air) = 100 mL/min.

As the active species in the ODS process, the effect of IL (C_16_PMoV) contents in the hybrid sample was investigated in the ODS process (Figure 5A). With a molar ratio (IL:Si = 1:30), the sulfur removal can only reach 28.6% desulfurization in 8 h. For the sample with the molar ratio = 1:20, the sulfur removal is increased to 82.6% under the same conditions, which cannot reach deep desulfurization. To further improve the IL content (molar ratio = 1:10), the desulfurization activity can achieve 96.4% in 5 h. Thus, the sample C_16_PMoV/10SiO_2_ was selected as the typical catalyst in the subsequent research to optimize the experimental conditions. The C_16_PMoV/10SiO_2_ sample was used as a typical catalyst to study the effect of mass and temperature on the desulfurization performance (Figure 5B,C). The activity of the catalyst is greatly influenced by both the quality of the catalyst and the reaction temperature, as shown in Figure 5B. Increasing the catalyst dosage can enhance the oxidation rate of sulfide. Notably, a significant rate enhancement is observed from 0.05 g to 0.06 g, while the further increase of the dosage to 0.07 g can slightly improve the catalyst’s efficiency. Therefore, the final catalyst selected was 0.06 g for environmental and economic considerations. Additionally, the reaction temperature can significantly affect the catalyst’s activity, as illustrated in Figure 5C. At 110 °C, the catalyst exhibits minimal desulfurization activity, indicating the inadequate activation of oxygen to produce free radicals. On the contrary, raising the reaction temperature to 120 or 130 °C can lead to a qualitative change in the catalyst’s activity, indicating the successful activation of oxygen into free radicals to promote the oxidative desulfurization process. Consequently, the final reaction temperature chosen was 120 °C. The optimized sulfur removal can reach 96.4% with 0.06 g of catalyst in 5 h at 120 °C.

**Figure 6:**
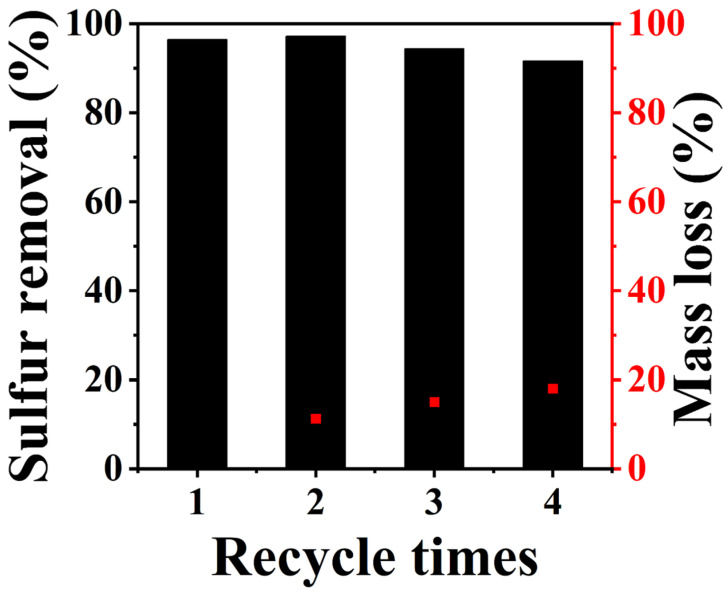
Recycling performance and mass loss of catalysts in oxidative desulfurization. Reaction condition: m(catalyst) = 0.06 g, T = 120 °C, ν(air) = 100 mL/min.

To investigate the ODS performance on different aromatic sulfides, the catalytic activity on DBT, 4-MDBT and 4,6-DMDBT was carried out under the same conditions (Figure 5D). From Figure 5D, the prepared sample exhibits a higher desulfurization efficiency on DBT compared to 4-MDBT and 4,6-DMDBT. The order of oxidative desulfurization (ODS) activity follows the order of DBT > 4-MDBT > 4,6-DMDBT. Consequently, the catalysts can exhibit effective activity on different types of sulfur-containing compounds. Therefore, the application of the catalysts can offer a promising solution for the challenging replenishment of complex sulfides after hydrodesulfurization. According to the previous study, the desulfurization rate is primarily influenced by the organic sulfide’s steric hindrance and the sulfur atom’s electron cloud density. Compared to 4,6-DMDBT and 4-MDBT, DBT has the lowest steric hindrance due to no methyl groups on the side chain, making the sulfide more easily oxidized. Additionally, the reusability and mass loss after circulation of the sample C_16_PMoV/10SiO_2_ were studied in this work (Figure 6). After each reaction, the remaining catalyst was separated, washed with carbon tetrachloride, and dried overnight at 60 °C. Afterwards, the used sample was weighed and then placed in a three-neck flask. Fresh oil was added, and the next round of operation was carried out at an air flow rate of 100 mL/min. It can be seen that the mass of the catalyst is continuously lost and the activity of the catalyst is gradually decreased. After recycling three times, the sulfur removal can reach 91.7%.

**Figure 7:**
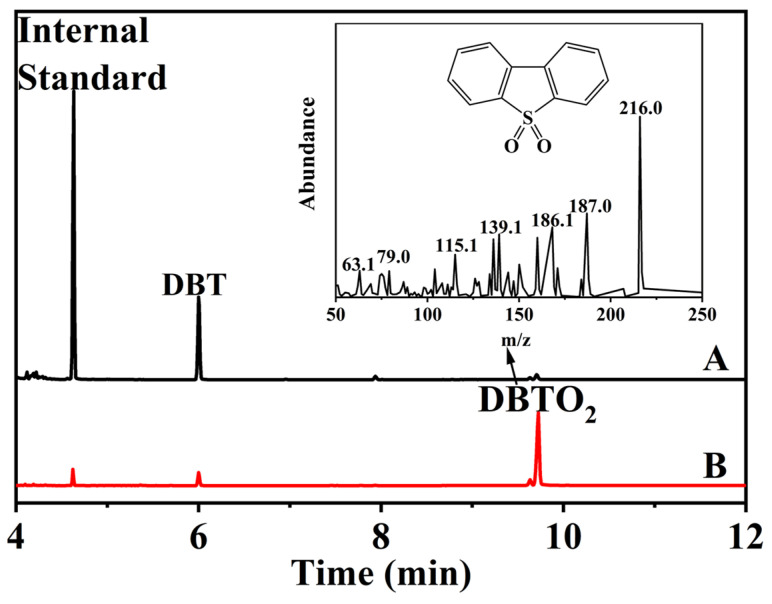
GC-MS analysis of the oil phase (A) and the catalyst phase (B) during the reaction. Reaction condition: m(catalyst) = 0.06 g, T = 120 °C, ν(air) = 100 mL/min.

To investigate the oxidative product in the ODS process, the oil phase and the catalyst phase during the reaction were determined by GC-MS analysis (Figure 7). After the reaction for 2 h, the oil phase was directly separated from the reaction system. In contrast, the catalyst was extracted by carbon tetrachloride. The peaks of the DBT (*m*/*z* = 186.1) and DBTO_2_ (*m*/*z* = 216) can be observed during the reaction both in the oil and catalyst phases. The results demonstrate that the catalyst can firstly adsorb DBT from the oil phase and then react with the oxidant (air) to produce free radicals that convert the adsorbed sulfide (DBT) to its corresponding sulfone (DBTO_2_).

**Figure 8:**
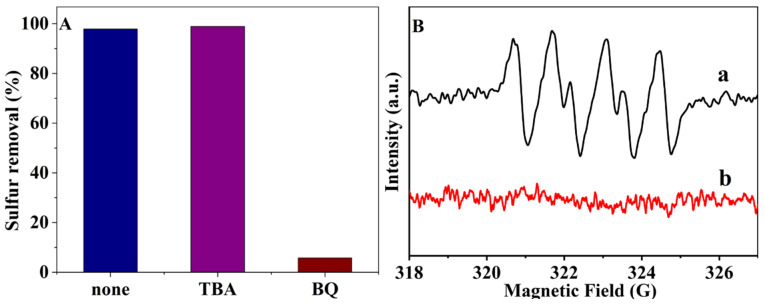
(**A**) The free radical capture experiment of catalyst C_16_PMoV/10SiO_2_; (**B**) ESR spectra of DMPO−•O_2_^−^ generated in the oxidation reaction of DBT with C_16_PMoV/10SiO_2_. (a) With a catalyst; (b) without a catalyst.

To study the active species in the ODS process, the free radical trapping experiment was conducted with p-benzoquinone (BQ) and tert-butanol (TBA) as the superoxide radical (•O_2_^−^) and hydroxyl radical (•OH) scavenger, respectively (Figure 8A). There was no significant change in the activity after the addition of TBA, indicating that the •OH did not have much effect in the reaction process. When BQ was added, the oxidative activity of the catalyst almost disappeared, and some of the remaining desulfurization rates may be due to the catalyst’s adsorption of sulfides in the fuel. These results suggest that the •O_2_^−^ radical is generated in the reaction, which can act as active sites in the ODS process. To further confirm the active site of •O_2_^−^ during the ODS process, an electron spin resonance (ESR) spin capture experiment was conducted using DMPO as a capture agent (Figure 8B). No unmistakable ESR signal can be detected without a catalyst, while a set of multi-fold peaks can be found, indicating that the catalyst was able to activate the incoming oxygen and thus generate superoxide radicals (•O_2_^−^). In conjunction with the GC-MS results, the catalyst firstly adsorbs sulfide and then activates oxygen to create free radicals to convert the adsorbed sulfide to the corresponding sulfone without the addition of an extractant.

## 3. Experimental Section

### 3.1. Materials and Methods

Dibenzothiophene (DBT, 98%) and 4,6-dimethyldibenzothiophene (4,6-DMDBT, 98%) were purchased from Sigma-Aldrich (Burlington, NJ, USA); 4-methyldibenzothiophene (4-MDBT, 96%), cetane (98%), molybdenum trioxide (MoO_3_, 99.9%) and fumed silica were purchased from Aladdin Chemical Reagent Co., Ltd. (Los Angels, CA, USA); dodecane and vanadium pentoxide (V_2_O_5_, 99.6%) were purchased from Shanghai Macklin Biochemical Technology Co., Ltd. (Shanghai, China); 1-hexadecyl-3-methylimidazole chloride salt ([C_16_mim]Cl, 99%) was procured from Shanghai Chenjie Chemical Co, Ltd. (Shanghai, China); acetonitrile (A.R. grade) and H_3_PO_4_ (85% wt%) were provided by Sinopharm Chemical Reagents Co., Ltd. (Shanghai, China).

The Fourier-transform infrared (FT-IR) spectra were measured with Nicolet Nexus 470 infrared spectrometer in the 400–4000 cm^−1^ region (KBr pellets) from Thermo Electron Corporation (Waltham, MA, USA). The samples’ X-ray diffraction (XRD) patterns were recorded on a Rigaku D/max 2500PC X-ray diffractometer in the 10~80° region with a scanning rate of 7°/min. Solid UV-vis diffuse reflection spectra (UV-vis DRS) were collected on a Japan Shimadzu UV-2450 UV-Vis spectrophotometer (BaSO_4_ discs) in the 200~800 nm region. The morphology of the various samples was observed by scanning electron microscopy (SEM) with a JSM-6010PLUS/LA, and the samples were coated with platinum to improve their electrical conductivity. X-ray photoelectron spectroscopy (XPS) on an ESCALAB 250Xi (Thermo Scientific K-Alpha, Waltham, MA, USA), in which the standard monochromatic Al Kα excitation was at 1361 eV, was utilized to determine the element valence of materials. The N_2_ adsorption-desorption isotherms were collected on a Thermo Fisher Micro 3020. X-band electron spin resonance (ESR) spectra were recorded at ambient temperature by a JES-FA200 (JOEL, Tokyo, Japan). The oxidized sulfur compound was characterized by GC-MS (Agilent 7890/5975C-GC/MSD; temperature program: 100 °C; temperature rising 15 °C min^−1^; 200 °C for 10 min; HP-5 MS column, 30 m × 250 μm i.d. × 0.25 μm).

### 3.2. Synthesis of Polyoxometalate H_8_PMo_7_V_5_O_40_

2.6 g of MoO_3_ and 1.2 g of V_2_O_5_ were dispersed in 65 mL of deionized water at room temperature, and the mixture was placed in an oil bath equipped with a condensing reflux device. Then, 0.5 mL of 85 wt% H_3_PO_4_ dissolved in 5 mL of deionized water was added dropwise to the mixture under continuous stirring at 100 °C for 24 h. After that, the mixture was cooled down to room temperature. Finally, H_8_PMo_7_V_5_O_40_ was obtained after freeze drying. 

### 3.3. Synthesis of Polyoxometalate Ionic Liquid 

At room temperature, 1.6 g of H_8_PMo_7_V_5_O_40_ was dissolved in 30 mL of deionized water. Then, 2.7 g of 1-hexadecyl-3-methylimidazolium chloride was added to the solution with continuous stirring for 8 h. Subsequently, the mixture was filtered and washed with deionized water and ethanol three times. Finally, the product was dried in a vacuum oven at 80 °C to obtain the polyoxometalate ionic liquid C_16_PMoV.

### 3.4. Synthesis of Polyoxometalate Ionic Liquid Supported Material 

A certain amount of C_16_PMoV and 0.5 g commercial grade SiO_2_ were added into the ball mill. The ball mill equipment was then operated at a speed of 200 rpm for 5 h in ambient air, and the rotation direction was altered every 5 min. Then, the targeted catalyst C_16_PMoV/*n*SiO_2_ (*n* = 10, 20, 30) was obtained, where *n* represented the molar ratio of Si/P in the sample.

### 3.5. Catalytic Activity Test

Model oil was acquired by dissolving the desired DBT, 4-MDBT, and 4,6-DMDBT in dodecane with a corresponding S-content of 200 ppm. The catalytic oxidative desulfurization was carried out in a three-necked flask connected to a reflux condenser and placed in a heating and temperature-controlled magnetic stirrer. Model oil (20 mL) and catalyst (60 mg) were added to the three-necked flask in a typical reaction. Air was added to the reaction system at a flow rate of 100 mL/min with continuous stirring; the stirring was turned off every hour, and when the stationary period was complete, 1 µL of the supernatant was taken to detect the current residual sulfur content. The residual sulfur content was tested via an internal standard method by gas chromatograph (Agilent7890A/5975C). The injector temperature was 300 °C, and the detector temperature was 250 °C. The temperature of the GC process started at 100 °C and rose to 200 °C at 15 °C/min and then rose to 250 °C at 20 °C/min. The residual sulfur concentration(*η*) in the model oil was calculated by the following equation:

$$\eta =\frac{C0-Ct}{C0}\times 100\%$$

*C*0 represents the initial S concentration of the model oil, and *Ct* represents the remaining S concentration of the upper oil phase after reaction time *t* after detection.

## 4. Conclusions

A series of hybrid materials based on polyoxometalate ionic liquid supported on SiO_2_ were successfully prepared via a facile ball milling method and successfully employed in the aerobic oxidative desulfurization process. The experimental results demonstrated that the ball milling process could successfully load the polyoxometalate ionic liquids on SiO_2_ without destroying the structure of the ionic liquids, and the active sites on the supported ionic liquids were uniformly dispersed on the support. The sample C_16_PMoV/10SiO_2_ exhibited high aerobic desulfurization activity on dibenzothiophene (DBT) in fuel, which can achieve a sulfur removal of 96.4% in 5 h with no organic solvents as an extractant. Moreover, the catalysts also exhibited good desulfurization activity for other various organic sulfides, and the desulfurization performance followed the order of DBT > 4-MDBT > 4,6-DMDBT. The oxidative product of DBT is considered as DBTO_2_ under the role of superoxide radical (•O_2_^−^), which was verified by the GC-MS analysis and free radical trapping experiment.

## Figures and Tables

**Table 1:** Textural properties of various samples.

Sample	S_BET_ (m^2^/g)	Pore Volume (cm^3^/g)	Average Pore Diameter (nm)
C_16_PMoV/10SiO_2_	256.7	0.67	1.51
C_16_PMoV/20SiO_2_	263.6	0.63	0.89
C_16_PMoV/30SiO_2_	339.7	1.02	0.88
SiO_2_	447.9	1.13	0.87

## Data Availability

The data presented in this study are available on request from the corresponding author.
